# Supplementary material for: Inhibition of the Receptor for Advanced Glycation End-Products in Acute Respiratory Distress Syndrome: A Randomised Laboratory Trial in Piglets
Source: Sci Rep. 2019 Jun 25;9:9227. doi: 10.1038/s41598-019-45798-5 (PMC6592897; doi:10.1038/s41598-019-45798-5)
Supplement: Supplementary file 1 — Supplementary Material [file 41598_2019_45798_MOESM1_ESM.docx]

**Supplementary Material**

**Inhibition of the Receptor for Advanced Glycation End-Products in Acute Respiratory Distress Syndrome: A Randomised Laboratory Trial in Piglets**

Jules Audard, Thomas Godet, Raiko Blondonnet, Jean–Baptiste Joffredo, Bertille Paquette, Corinne Belville, Marilyne Lavergne, Christelle Gross, Justine Pasteur, Damien Bouvier, Loic Blanchon, Vincent Sapin, Bruno Pereira, Jean–Michel Constantin, Matthieu Jabaudon

| **Parameter** | **0** | **1** | **2** |
| --- | --- | --- | --- |
| **i. Neutrophils in the alveolar space** | None | 1–5 | >5 |
| **ii. Neutrophils in the interstitial space** | None | 1–5 | >5 |
| **iii. Hyaline membranes** | None | 1 | >1 |
| **iv. Proteinous debris filling the airspaces** | None | 1 | >1 |
| **v. Alveolar septal thickening** | None | 2×–4× | >4× |

**Table 1. Lung Histology Injury Scoring System** (adapted from Matute-Bello et al. [[33]](https://paperpile.com/c/8s4L2w/mkBxb))**.**

|  | **Sham** | **HCl** | **HCl+RAP** | **HCl+sRAGE** | **P**  **for time × group interaction** | **P**  **for post–hoc comparisons** |
| --- | --- | --- | --- | --- | --- | --- |
| **Tidal Volume (mL.kg^–1^)**  Baseline  H1  H2  H3  H4 | 6.5 [6.3–6.6]  6.5 [6.3–6.6]  6.5 [6.3–6.6]  6.5 [6.3–6.6]  6.5 [6.3–6.6] | 6.4 [6.3–6.5]  6.4 [6.3–6.5]  6.3 [6.2–6.5]  6.3 [6.3–6.4]  6.3 [6.2–6.5] | 6.6 [6.5–6.6]  6.6 [6.5–6.6]  6.6 [6.5–6.6]  6.6 [6.5–6.6]  6.6 [6.6–6.6] | 6.5 [6.1–6.7]  6.5 [6.1–6.7]  6.5 [6.1–6.7]  6.5 [6.1–6.7]  6.5 [6.1–6.7] | 0.9 |  |
| **Pplat (cmH_2_O)**  Baseline  H1  H2  H3  H4 | 16 [15–17]  16 [13–17]*  15 [12–17]*  16 [13–20]*  15 [13–19]* | 15 [14–16]  20 [18–24]*^,^**  21 [19–25]*^,^**^,^***  23 [22–25]*^,^**^,^***  21 [19–25]*^,^**^,^*** | 15 [13–17]  17 [16–19]  15 [14–17]**  15 [13–17]**  17 [14–18]** | 16 [14–17]  16 [13–19]**  16 [15–19]***  14 [13–17]***  15 [13–17]*** | <10^–4^ | NS  *P = 0.001, **P = 0.01  *^,^**P <10^–4^, ***P = 0.001  *^,^**^,^***P <10^–4^  *^,^***P <10^–4^, **P = 0.001 |
| **C_RS_ (mL.cmH_2_O^–1^)**  Baseline  H1  H2  H3  H4 | 6.2 [5.2–7.0]  6.1 [5.6–8.4]*  6.5 [5.2–9.5]*  5.8 [4.7–9.2]*  6.4 [5.1–8.2]* | 6.8 [6.5–7.8]  4.9 [3.5–5.0]*  4.4 [3.0–5.2]*  3.6 [3.3–4.6]*^,^**^,^***  4.6 [3.3–4.9]*^,^** | 6.8 [5.3–8.1]  5.6 [4.5–6.1]  6.4 [5.1–7.4]  6.8 [5.3–9.1]**  5.5 [4.7–7.2] | 6.1 [5.0–6.8]  5.6 [4.5–7.6]  5.6 [4.6–6.2]  6.4 [5.2–8.0]***  6.4 [5.1–8.0]** | <10^–4^ | NS  *P = 0.04  *P = 0.008  *P = 0.004, **P = 0.001, ***P = 0.005  *P = 0.002, **P = 0.005 |
| **ΔP (cmH_2_O)**  Baseline  H1  H2  H3  H4 | 11 [10–12]  11 [8–12]*^,^**  10 [7–12]*  11 [8–15]*  10 [8–14]* | 10 [9–11]  15 [13–19]*  16 [14–20]*^,^**^,^***  18 [17–20]*^,^**^,^***  16 [14–20]*^,^**^,^*** | 10 [8–12]  12 [11–14]  10 [9–12]**  10 [8–12]**  12 [9–13]** | 11 [9–12]  11 [8–14]**  11 [10–14]***  9 [8–12]***  10 [8–12]*** | <10^–4^ | NS  *P = 0.0002, **P = 0.003  *^,^**P <10^–4^, ***P = 0.0009  *^,^**^,^***P <10^–4^  *^,^***P <10^–4^,** P = 0.0007 |
| **PaCO_2_ (mmHg)**  Baseline  H1  H2  H3  H4 | 47 [44–49]  46 [43–52]  47 [41–53]  45 [41–50]  47 [41–50] | 49 [36–58]  56 [45–61]  52 [43–65]  56 [41–73]  59 [41–79] | 41 [37–47]  46 [44–50]  47 [45–51]  50 [48–51]  48 [45–52] | 44 [39–50]  51 [44–60]  49 [42–52]  49 [41–56]  48 [44–58] | 0.07 |  |
| **Arterial pH**  Baseline  H1  H2  H3  H4 | 7.32 [7.29–7.35]  7.33 [7.28–7.35]  7.31 [7.29–7.36]*  7.34 [7.29–7.37]*  7.35 [7.28–7.37]* | 7.32 [7.18–7.46]  7.22 [7.11–7.29]  7.20 [7.13–7.31]*  7.19 [7.06–7.31]*^,^**  7.16 [7.04–7.31]*^,^**^,^*** | 7.38 [7.33–7.40]  7.27 [7.26–7.30]  7.28 [7.25–7.30]  7.26 [7.22–7.28]**  7.27 [7.24–7.30]** | 7.35 [7.30–7.41]  7.27 [7.22–7.32]  7.28 [7.25–7.31]  7.29 [7.24–7.35]  7.28 [7.23–7.36]*** | <10^–4^ | NS  NS  *P = 0.04  *P = 0.001, **P = 0.03  *P = 0.001, **P = 0.04, ***P = 0.02 |
| **MAP (mmHg)**  Baseline  H1  H2  H3  H4 | 52 [50–58]  52 [48–62]  51 [50–55]  51 [50–55]  54 [51–60] | 62 [47–67]  59 [54–63]  58 [51–61]  58 [51–61]  62 [53–67] | 53 [51–56]  55 [52–60]  55 [53–60]  55 [53–60]  59 [51–65] | 58 [52–64]  56 [53–62]  55 [58–62]  55 [58–62]  55 [58–64] | 0.8 |  |
| **Cardiac Index (L.min^–1^.m^–2^)**  Baseline  H1  H2  H3  H4 | 3.2 [2.9–3.6]  3.4 [3.0–3.9]  3.3 [3.0–3.5]  3.3 [3.0–3.5]  3.4 [3.1–3.6] | 3.1 [2.6–3.9]  3.4 [2.8–4.2]  3.4 [2.8–4.0]  3.4 [2.8–4.2]  3.7 [3.0–4.1] | 3.0 [2.7–3.4]  3.1 [2.8–4.1]  3.3 [2.8–4.1]  3.3 [2.8–4.1]  3.1 [2.6–3.4] | 4.2 [3.9–5.0]  4.1 [3.6–4.8]  4.2 [3.3–4.7]  4.1 [3.3–4.6]  3.6 [2.6–4.7] | 0.1 |  |
| **Serum Lactate (mmol.L^–1^)**  Baseline  H1  H2  H3  H4 | 4.9 [3.4–5.5]  4.9 [3.5–5.4]  4.9 [3.1–5.4]  5.2 [3.1–5.4]  5.1 [3.1–5.3] | 5.2 [3.0–6.1]  6.0 [4.3–7.0]  6.3 [5.2–7.2]  6.8 [5.5–7.6]  6.4 [5.0–7.6] | 5.6 [4.6–7.2]  6.6 [5.0–7.9]  6.8 [5.3–8.1]  7.2 [5.5–8.0]  6.9 [5.5–8.8] | 4.5 [3.4–7.2]  5.3 [3.6–7.9]  5.8 [3.7–8.0]  5.5 [4.4–8.1]  5.2 [4.3–7.7] | 0.1 |  |

**Table 2. Respiratory and Hemodynamic Parameters.** Data are presented as medians and interquartile ranges [IQR] and are analysed with two-way repeated-measurement analysis of variance. When significant, Mann-Whitney test (nonparametric data) was used for post–hoc comparisons between groups at each time point.

*Pplat: inspiratory plateau pressure. C_RS_: compliance of the respiratory system. ΔP: driving pressure. PaCO_2_: arterial carbon dioxide tension. MAP: mean arterial pressure. NS: non-significant.*
